# Human infants can override possessive tendencies to share valued items with others

**DOI:** 10.1038/s41598-021-88898-x

**Published:** 2021-05-05

**Authors:** Rodolfo Cortes Barragan, Andrew N. Meltzoff

**Affiliations:** 1 Institute for Learning and Brain Sciences, University of Washington, Seattle, USA; 2 Department of Psychology, University of Washington, Seattle, USA

**Keywords:** Psychology, Human behaviour

## Abstract

Possessiveness toward objects and sharing are competing tendencies that influence dyadic and group interactions within the primate lineage. A distinctive form of sharing in adult *Homo sapiens* involves active giving of high-valued possessions to others, without an immediate reciprocal benefit. In two Experiments with 19-month-old human infants (*N* = 96), we found that despite measurable possessive behavior toward their own personal objects (favorite toy, bottle), infants spontaneously gave these items to a begging stranger. Moreover, human infants exhibited this behavior across different types of objects that are relevant to theory (personal objects, sweet food, and common objects)—showing flexible generalizability not evidenced in non-human primates. We combined these data with a previous dataset, yielding a large sample of infants (*N* = 192), and identified sociocultural factors that may calibrate young infants’ sharing of objects with others. The current findings show a proclivity that is rare or absent in our closest living relatives—the capacity to override possessive behavior toward personally valued objects by sharing those same desired objects with others.

## Introduction

To different degrees, all primate species show possessive behavior toward objects starting from early in development^1–3^. This behavior can be balanced against a countervailing tendency that often overrides it—the sharing of objects with others^4–6^. Human adults are notable for their possessiveness and also for sharing items with non-kin strangers. There are considerable individual differences, with some people displaying hoarding of objects and limited sharing^7–9^. Others display little possessiveness and readily share things that belong to them, even items of significant value^10,11^. There are also cultural differences in the balance between the tendency for possessiveness versus sharing in humans^12,13^. Active sharing behavior with non-kin, across many types of objects and in the absence of immediate reciprocity, is distinctive to human adults compared to other primates^6,14^.

What about human infants? Studies on language development provide a starting point: Many infants produce possessive language (e.g., “mine” or “yours”) and act possessively toward specific items by 18 months of age^15^—an ability that is evident in most infants by about 24 months of age^16,17^. Weighing against this possessiveness, infants also act prosocially and selectively share objects with adults under certain conditions^18–21^. This was demonstrated experimentally in a pioneering laboratory study designed to test infants and chimpanzees (18 and 36–54 months old, respectively)^22^. In one task used in this study, an adult showed the infant that he was trying to hang towels, but the clothespin slipped from the adult’s hand, and the behavioral measure was whether the infant would hand the clothespin back to the adult. Using variants of this accidental-dropping paradigm, researchers have reported that infants help or actively share a variety of common objects with both kin and non-kin strangers^23–25^, whether there is explicit reward or not^26^, and with both familiar and unfamiliar items^27,28^.

There have also been efforts to test whether infants will share objects that they may wish to possess. One study evaluated whether infants would share objects brought from home, when exposed to a multistep series of increasingly explicit cues (e.g., experimenter progressing from shivering to saying “I need something to make me warm” to asking the child “Can you bring me the blanket?”)^29^. A follow-up study with 16- to 36-month-olds replicated this multistep procedure in the context of providing the parents with items for use at home and then testing the children a week later; results showed that children sometimes gave the object following prompts such as “Can you give me the scarf?”^30^. (The Supplementary Information provides more details about this line of work.)

Nonetheless, it has not yet been shown what infants will do when there is a direct conflict between retaining possession of a high-valued object versus actively giving that same object to someone who shows that they also want it. This, of course, is what many adults do in day-to-day life: Across a variety of objects, adults in a range of cultures will spontaneously give up objects of high personal value (food, money, personal possessions) in favor of giving them to others, even strangers^31,32^. Some researchers have characterized this as an aspect of humans’ hyper-cooperativity and a building block of human morality^6,8,28,33,34^. This flexible sharing of valued personal objects with strangers, even in cases without reciprocity, has been understudied in infants. In light of this gap, we sought a non-verbal procedure that could be used to measure both infant possessiveness and sharing in the same setting, and which might be adaptable for use with non-human primates to inform comparative and developmental psychology questions about commonalities/distinctiveness in “sociality profiles” among human adults, human infants, and non-human primates^35–38^.

What type of objects do human infants value to such an extent that we could evaluate their overriding wanting-for-the-self in favor of prosocial sharing? There is a particular class of things that human infants seek out as highly valued. This class has often been dubbed “attachment”^39^, “transition”^40^, or “contact comfort”^41^ objects. Common exemplars are their own stuffed teddy bear or blanket. In naturalistic observations, it has been noted that infants often lean/lunge towards or try to possessively grab these treasured objects^39^.

Building on previous research with human infants^22–28^, we conducted two interlocking experiments on the nature, scope, and flexibility of infants’ giving away of high-value objects to others. The accidental-drop procedure we adopted was designed to exclude linguistic prompts (such prompts make comparisons with non-human primates difficult), exclude immediate reciprocity by the adult, and to provide infants with an escape route which made it easy for them to grab their high-value object and retreat to their parent, rather than to share with the experimenter. Experiment 1 provided an initial test; Experiment 2 used a range of objects of different value to the infants, examined infants’ responses to these objects, and introduced a measure of infant possessive behavior. Finally, we combine across the experiments and explore the malleability of human infant sharing. We report a capacity for sharing personally valued items that is simultaneously present in human infancy and also influenced by the infants’ own sociocultural experiences.

## Experiment 1

### Results

In this experiment we asked parents to bring to the laboratory two of the infant’s own treasured objects: (a) the infant’s own favorite toy and (b) the infant’s own bottle/cup that they routinely drank from (often called a “sippy cup” because infants at this age can suck liquids through a plastic spout). Typically, others in the family do not use the infant’s bottle/sippy cup, because it “belongs” to the child^40,42^. During the consenting process, and out of view of the infant, parents surreptitiously put the favorite toy and bottle into a pouch and slipped them to the experimenter prior to the start of the experiment.

The participants were 48 infants who were 19-months old. The experiment took place at the university in a laboratory and was digitally recorded for subsequent blind coding. We adapted an accidental-drop procedure in which the adult is seen to either accidentally drop an item (Experimental group) or to intentionally toss it to the same spot on the floor (Control group) to assess whether or not the infant picks up the item and actively gives it to the adult^22,28^. In the Experimental group, an experimenter sitting across the table from the infant fumbled the objects such that they seemed to accidentally fall from his hand, landing out of his reach on the floor on the infant’s side of the table. The experimenter unsuccessfully reached for the fallen object but was blocked by the table. A fixed, 20-s response period was electronically timed, during which the experimenter exhibited a pleasant but neutral facial expression independent of infants’ actions. During this period there were no linguistic instructions provided to infants (see Methods). Thus, infants did not receive multistep linguistic requests for assistance as is sometimes the case in related studies in the human infant literature^29,30^ (Supplementary Information).

The majority of the favorite toys brought in for the test were soft objects and had rostral features resembling the characteristics that Lorenz^43^ termed *Kindchenschema* or baby-facedness. These personal objects were predominantly stuffed animals (58.33%) and dolls (10.42%) and also included vehicles (6.25%), plastic toys (6.25%), blankets (4.17%), books (4.17%), balls (2.08%), and miscellaneous items (8.33%). Preliminary analyses showed that the counterbalanced factors in this experiment were not significant: Specifically, there were no significant main effects or interactions for test order or gender of the infant, and therefore, analyses are presented collapsed across those factors.

**Figure 1:**
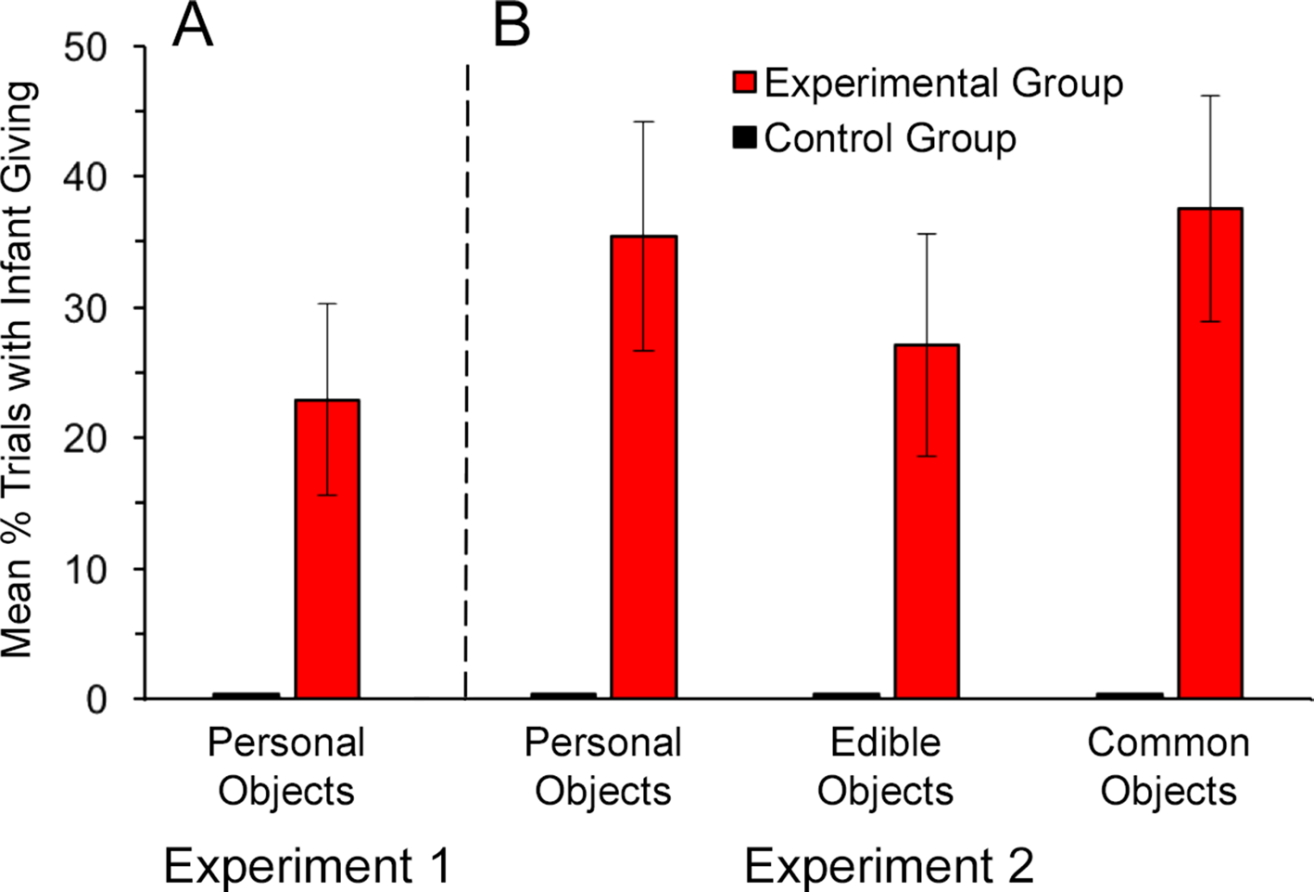
Mean percent of trials with active giving behavior in Experiment 1 and Experiment 2, by object type. All comparisons between the Experimental (red) and the Control (black) groups are significant (permutation tests). (**A**) Experiment 1: *z* = 2.86, *P* = 0.004, *r* = 0.41. (**B**) Experiment 2: personal objects: *z* = 3.51, *P* = 0.0002, *r* = 0.51; edible objects: *z* = 2.91, *P* = 0.004, *r* = 0.42; and common objects: *z* = 3.69, *P* = 0.00008, *r* = 0.53. Error bars show standard errors.

The results showed that 33.33% (8/24) of the infants in the Experimental group picked up at least one of these personal items and handed it to the experimenter, versus 0% (0/24) of infants in the Control group, *P* = 0.004, Φ = 0.45, Fisher’s exact test, two-tailed (all statistics are reported using two-tailed tests). The mean percent of trials in which infants gave the items to the adult was significantly greater in the Experimental group (*M* = 22.92%, *SD* = 36.05) than in the Control group (*M* = 0%), *z* = 2.86, *P* = 0.004, *r* = 0.41 (permutation test) (Fig. 1A).

These patterns show that by 19 months of age, some human infants, not the majority, actively give their personal items to a begging stranger. However, there was no direct comparison to non-personal objects, which became the focus of Experiment 2 and allowed us to measure the extent of infants’ expression of desire or possessiveness toward their personal objects.

## Experiment 2

This experiment tested a wider range of objects than were used in Experiment 1, and the types of objects selected were driven by theory. We used three salient classes of objects in infants’ lives: personal objects (favorite toy and bottle), highly desirable edible objects (slice of banana and half of a grape), and common objects (wooden cube and marker). This allowed us to assess two key issues in the comparative and developmental psychology literatures—object value and flexibility of object sharing across different items.

First, we explored “value” by assessing whether infants displayed more possessive behavior toward their personal objects than toward objects that do not belong to them. Heretofore, studies on giving among non-human primates and human infants did not measure the value that the test object had for the subjects. We were able to index value by introducing non-verbal measures of the desire for the objects tested, which involved measurements of infant lunging toward them and reaching out with their hands when the experimenter first showed them to the infant during the test (see Method). Previous studies using objects in infant object sharing paradigms have not had assessments of how much infants wanted the objects in the experiment and therefore the balance between self-wanting and sharing with another person was not assessed.

Second, Experiment 2 takes on importance because active sharing by human adults is flexible and generalizes across types of objects. While adults actively give a range of objects to non-kin, the sociality profile of non-human primates is such that chimpanzees (*Pan troglodytes*) tend to give common objects to others, but not food^22^, and bonobos (*Pan paniscus*) tend to give food and not common objects^14,36^. We assessed the flexibility of human infants, employing a repeated-measures design with the same infants tested on six items (two personal objects, two food objects, and two common objects). We examined both whether infants showed elevated signs of possessiveness toward their own personal objects versus the other objects tested and the degree to which infants actively give different types of objects (which would assess the flexibility demonstrated by adults and not typically observed in non-human primates).

### Results

Preliminary analyses showed that neither of the counterbalanced factors (gender, test order) was significant, and therefore analyses are presented collapsed across those factors. The infant treasured objects brought in by the parents were: stuffed animals (64.58%), dolls (4.17%), vehicles (6.25%), plastic toys (4.17%), blankets (4.17%), books (6.25%), balls (4.17%), and miscellaneous items (6.25%).

**Figure 2:**
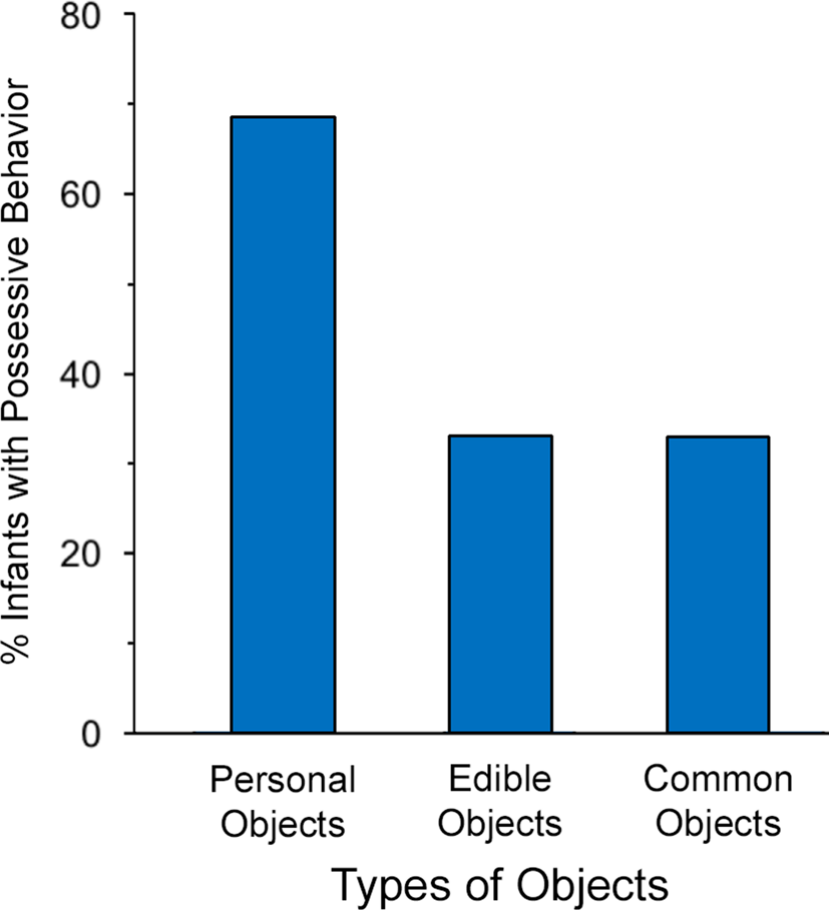
Percent of infants exhibiting possessive behavior as a function of object type in Experiment 2. More infants displayed possessive behavior (dichotomous yes/no) toward their own personal objects than to edible objects or common objects (see text for statistical details).

**Figure 3:**
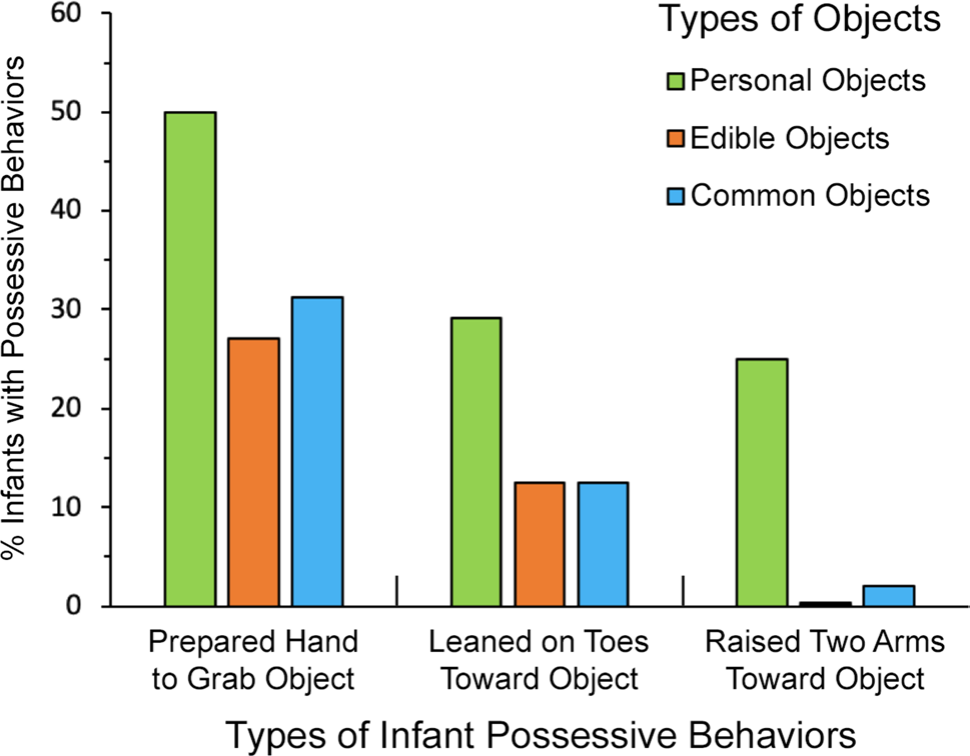
Percent of infants exhibiting different types of possessive behavior for personal objects (green), edible objects (orange), and common objects (blue) in Experiment 2. Each infant was scored for possessive behaviors (dichotomous yes/no) shown toward the objects. Three different kinds of infant possessive behaviors were scored (x-axis). For each type of infant possessive behavior, significantly more infants produced it for personal objects than for edible objects and common objects. This was assessed statistically using Cochran *Q* (2, *N* = 48) tests. (a) Prepared hand to grab object: *Q* = 7.92, *P* = 0.019, η^2^_Q_ = 0.08. (b) Leaned on toes toward object: *Q* = 7.53, *P* = 0.023, η^2^_Q_ = 0.08. (c) Raised two arms toward object: *Q* = 22.17, *P* = 0.00002, η^2^_Q_ = 0.23.

As predicted, the number of infants exhibiting possessive behavior (dichotomous yes/no) significantly varied as a function of object type, *Q*(2, *N* = 48) = 20.64, *P* = 0.00003, η^2^_Q_ = 0.22 (Cochran test), and was highest for the personal objects. As shown in Fig. 2, a majority, 68.75% (33/48) of infants, directed possessive behavior toward a personal object compared to 33.33% (16/48) toward an edible object and 33.33% (16/48) toward a common object. More fine-grained analyses showed that each of three kinds of possessive behaviors was significantly elevated in response to the personal objects (Fig. 3). We also went back to Experiment 1 to check whether the level of possessive behavior shown to the infant’s own personal objects was similar across the two studies. Indeed, the level of possessive behavior was stable: There were 29/48 infants in Experiment 1 who showed possessive behavior to their personal objects versus 33/48 infants in the new sample of infants in Experiment 2, χ^2^ (1, *N* = 96) = 0.41, *P* = 0.52, Φ = 0.09, suggesting that infants produce a replicable (high) level of possessiveness toward their personal objects.

Next, we analyzed infants’ active giving. Results showed that a majority, 62.50% (15/24) of the infants, in the Experimental group picked up at least one of the six objects and handed it to the adult, versus 0% (0/24) of the infants in the Control group, χ^2^(1, *N* = 48) = 19.01, *P* = 0.00001, Φ = 0.67. This difference between the experimental and control infants was also significant for each of the three categories of objects: personal objects, 45.83% (11/24), χ^2^(1, *N* = 48) = 11.79, *P* = 0.0006, Φ = 0.55 (replicating Experiment 1), edible objects, 33.33% (8/24), *P* = 0.004, Φ = 0.45, Fisher’s exact test, and common objects, 50.00% (12/24), χ^2^(1, *N* = 48) = 13.44, *P* = 0.0002, Φ = 0.58. The mean percent of trials in which infants picked up and transferred an object was greater in the Experimental group (*M* = 33.33%, *SD* = 37.75) than in the Control group (*M* = 0%), *z* = 3.69, *P* = 0.000002, *r* = 0.53 (permutation test). This significant effect was also obtained with each type of object (personal, edible, common) considered individually (all *P*s ≤ 0.004), see Fig. 1B, with no significant difference in giving in the Experimental group across the types of objects, *P* = 0.31 (permutation test).

## Statistical patterns across the two experiments

Next, we analyzed patterns that can be assessed with the larger sample of infants (*N* = 96) by pooling the data from Experiment 1 and 2. We report five analyses that highlight the degree to which human infants can overcome possessiveness in favor of sharing with non-kin strangers.

First, there is strong evidence that infants give even when it involves their highly valuable objects: 39.58% (19/48) of the infants in the Experimental group gave their personal items, and 0% (0/48) of the infants in the Control group did so, χ^2^(1, *N* = 96) = 21.26, *P* = 0.000004, Φ = 0.50. The mean percent of trials with personal object transfer was significantly greater in the Experimental group (*M* = 29.17%, *SD* = 39.72) than in the Control group (*M* = 0%), *z* = 4.53, *P* = 0.0000004, *r* = 0.46 (permutation test).

Second, within the Experimental group, the number of infants who give the experimenter their bottle (15/48) did not significantly differ from the number who gave him their favorite toy (13/48), χ^2^ (1, *N* = 48) = 0.10, *P* = 0.75 (McNemar test), showing that infants were willing to share their personal items at similar rates.

Third, we analyzed whether infants who show possessive behavior with their personal object during the adult’s demonstration period also subsequently actively gave that object of desire. Across the two experiments, a total of 62 infants showed possessive behavior toward their personal objects (favorite toy or bottle). The number of infants who exhibited such possessive behavior was evenly distributed between the Experimental (31 infants) and Control (31 infants) groups, as expected by chance and random assignment. We analyzed whether infants who showed possessive behavior toward a personal object (dichotomous yes/no) went on to give the adult that same object. The results showed that 32.26% (10/31) of the infants in the Experimental group did so in contrast to 0% (0/31) in the Control group, χ^2^(1, *N* = 62) = 9.66, *P* = 0.002, Φ = 0.44. This indicates that even if we focus only on those particular infants who exhibited overt possessive behavior toward a specific personal object (i.e., evidenced a desire for it), some of these infants could nonetheless engage in active giving with that same object.

Fourth, sleeping with an object is a marker of an emotional investment^44^, and parents reported that 62 infants (30 and 32 respectively in the Experimental and Control groups) slept with their treasured personal item at least some of the time. An analysis of these infants showed that 30.00% (9/30) of the infants in the Experimental group who slept with their item gave the experimenter this object, compared to 0% (0/32) of the infants in the Control group, *P* = 0.0007, Φ = 0.43, Fisher’s exact test. These results further suggest that active giving occurred with specific objects that are important to infants in the course of their everyday lives, including during the night.

Finally, we checked to confirm that infants in the Experimental group did not see the experimenter possess infants’ objects for any longer than infants in the Control group (i.e., during the demonstration period before the object was released to the tray on the floor). Specifically, we analyzed the duration of experimenter’s holding the item before release, and it was brief (~ 5 s) and not significantly different between groups: Experimental group (*M* = 5.24 s, *SD* = 0.80) versus Control group (*M* = 5.56 s, *SD* = 1.19), *z* = 1.51, *P* = 0.13, *r* = 0.15 (permutation test).

## Social-experiential predictors

It is of interest for theory to explore whether prior sociocultural experiences^45^ might predict the expression of infant sharing. In previous work^28^ using a different sample of *N* = 96 infants of the same age as tested in this paper, we found that having siblings and certain parental ethnic-cultural backgrounds (i.e., Asian and Hispanic/Latino) was associated with infant sharing behavior in the laboratory. This was of interest because Asian and Hispanic/Latino cultures emphasize the value of the group over individual needs^45–47^. We combined the available data from across the two papers (*N* = 192) to examine these sociocultural variables, after preliminary analyses indicated that there were no significant effects attributable to Paper (previous vs. current paper) and no Paper × Experimental group interaction, *P*s > 0.52 (both studies were conducted by the same experimenter, in the same test room, and used similar procedures).

We analyzed these data using a two-step hierarchical regression using the percent of trials with giving as the criterion (Table 1). In Step 1 of the regression, we entered Experimental group, and in Step 2 we entered siblings and ethnic-cultural background as socialization variables. The overall model explained 27.24% of the variance. Experimental group accounted for 20.81% of the variance (*P* = 3 × 10^−11^), and the socialization variables together accounted for an additional 6.43% of the variance (*P* = 0.0003), with each exhibiting a significant effect. Specifically, after controlling for other variables in the model, infants of Asian and/or Hispanic/Latino backgrounds had, on average, 22.96% more active giving across trials than infants of other ethnic-cultural backgrounds, and infants with siblings had, on average, 8.97% more active giving across trials than infants without siblings. This pattern suggests a relation between prior sociocultural experiences at home and infant behavior in the experiment, over and beyond group assignment. Future work using experimental interventions on infant interactions at home^5^ would be useful, as would a deeper analysis of individual caregiving differences within ethnic-cultural groups. Building on the correlational data provided here, designed interventions would allow tests of causal links between specific socialization experiences and the expression of sharing behavior to a non-kin stranger in the laboratory.

**Table 1:** Summary of hierarchical multiple regression analysis with percent of trials with active giving as criterion (*N* = 192). Because the criterion had a limited number of possible values and groups displayed different variances, we also modeled the data using RLM macro, version 1.01^92^, with HC = 3-type robust standard errors. The results using robust standard errors^93^ remained substantively the same both in Step 1 and Step 2, with all predictors remaining *P* < 0.05.

Regression step	Predictor variable	*b*	*SE*	*t*	Δ*R*^2^	*P*
Step 1	Group	28.91	4.09	7.07	0.208	0.00000000003
Step 2				2.88	0.064	0.0003
	Siblings	8.97	4.14	2.17		0.032
	Ethnic-cultural background	22.96	6.78	3.38		0.0009

## General discussion

We found that human infants, by 19-months of age, show significant possessive behavior to their personal items and that some infants can override this possessiveness in favor of giving up a high-value object to a non-kin stranger. Moreover, sociocultural variables predicted the degree of giving observed in the laboratory.

We designed the experiments in specific ways that could have tempted the infant to selfishly seize the valuable objects for themselves. That is, the room was arranged such that the experimenter was seated behind a table when presenting the objects, and the infant was on the other side of the table at a short distance from their parent. In order to take his seat at the table the experimenter had to open and close a gate, and once in place he did not have direct spatial access to the infant (see Methods). This arrangement was used so that the infants could pick up their objects and easily escape directly to their parent instead of giving up their objects to the stranger. Finally, the experiment was set up so there was no extrinsic reward or reciprocal gift: The adult simply accepted the object with a neutral facial expression and put it below the table—out of sight of the infant.

The results shed light on the commonalities and distinctions in human infants’ possessiveness and active sharing compared to related behaviors shown by other primates. Chimpanzees exhibit a tendency to engage possessively toward objects such as stone-hammers^48^, sticks^49^ and easily monopolizable food^1,6^. Despite this, chimpanzees show an ability to give beggars certain low-value objects such as blocks and sponges, but not food^22^. By contrast, bonobos are less ready to give common objects but do give food to others^36^—although this is most readily manifest in circumstances when they obtain benefits for themselves (e.g., social contact^50^). We know of no experiment that has examined active giving by chimpanzees or bonobos which involved the animal’s own personal, favorite objects. The possibility of cross-species comparisons with favorite objects has been somewhat limited up to now, because previous infant human tests on the topic have involved explicit verbal prompts directed to the children (e.g., saying, “Can you help me?” or “Can you give me the [object]?”)^29,30^, which is a different procedure than can be used to test non-human primates^22,36^, or was used here.

The current findings invite future experiments with chimpanzees and bonobos that use their personally favorite objects. Based on the extant literature^1–6,14,36^, we suggest that other non-human primate species are unlikely to succeed across a range of objects (personal objects for which they have demonstrated possessiveness, desirable food, common objects), particularly in the absence of immediate reciprocity and the presence of an escape route allowing subjects to grab the objects and keep them for themselves. The flexible expression of this sharing of personally valued items may be distinctively human. Yet, it is manifest in human infants by 19 months of age.

Although the findings are intriguing, there are issues of interpretation that would benefit from further work. It is possible that in the experimental group, after the adult accidentally drops the object, the adult’s begging gesture could have been interpreted as a command to return the desirable object to him, and thus the act of picking up and giving the objects could be motivated by obedience or docility, rather than prosocial sharing. Yet, compliance with social norms is itself a type of prosociality^51^. The potential distinction between sharing versus compliance (or obedience) may be challenging to measure in nonverbal infants and other primates. Nonetheless, it is of interest to theory^6^, and some research has begun to examine infants’ representations of obedience^52,53^. Future research might attempt to design studies to explore how finely-differentiated infants’ representations might be with respect to prosocial sharing, compliance, and/or obedience to tease out the specific motives for the observed infant giving behavior.

Our investigation also points to further work on infants’ assessment of the value of objects. While our coding of possessiveness introduced a measurement of the non-verbal desire to possess objects, this did not tap infants’ neural processing of the events as they unfolded^54^, nor infants’ emotional reactions. We do not know if infants weighed the value of the object during the moment of giving, but we know that infants showed possessive behavior, sometimes lunging toward their personal objects when they saw them. It would be interesting to examine whether giving away a treasured toy induced a cost^28,55^, particularly an affective, “emotional cost” that may have been undetectable by our behavioral coding. Future research, perhaps using infant emotional expressions, pupil dilation^56^, physiological assessments (e.g., heart rate^57^), or brain measures might be able to detect whether infants experience a greater emotional cost in sharing their personal objects than they do sharing common objects (despite similar performance on our behavioral measure). Also of interest is whether infants in our study might have experienced a self-rewarding positive emotion after acting in a generous, sharing fashion, characterized as feeling of “warm glow” in adults and older toddlers^58,59^. As such, it remains to be investigated how infants affectively, neurally, and cognitively process situations in which they give away their own items—which they may desire to possess—to non-kin strangers.

The current findings connect to broader theories in social and developmental psychology^6,8,11,60–62^, by pointing to a role for early life history and sociocultural experiences in the expression of human prosocial behavior. The current infant data suggest that already by 19-months of age, certain sociocultural experiences are associated with infant sharing. We acknowledge that these results do not permit causal inferences, but do invite discussion about possible developmental mechanisms^63–65^.

Siblings afford children with multiple opportunities for learning that others have desires and intentions directed toward objects^66^. Longitudinal research involving naturalistic home observations and analyses using birth-order suggests that siblings afford children with opportunities to practice prosocial interactions^67^, including opportunities for coordinated social engagement^68^ and reciprocal play^69^. Experimentally induced reciprocal play between adults and infants has been reported to increase infant helping behavior^70,71^, and so it is not implausible that experiences of reciprocal play with siblings could also play this role.

What about the ethnic-cultural effects? In the U.S. context, different cultural groups have measurably different socialization values and practices^72,73^, and while we cannot specify which particular family value may be at play here, we can offer several possibilities. Parents of East Asian heritage teach values similar to Japanese *sunao* (open-hearted cooperation), and research has shown that East Asian children are motivated to adopt parental cultural customs and norms^45,74^. Relatedly, from a young age, U.S.-based Hispanic/Latino children are exposed such cultural values as *acomedido* (to be attentive to others’ needs and help without being asked)^75^ and *simpátia* (to be gregarious and hospitable)^76^. In interactions with toddlers, Mexican immigrant mothers emphasize the tendency toward positive demeanor^77^. Furthermore, Hispanic/Latino and Asian children may be socialized to value the needs or wants of others and/or to put less value on retention of objects solely for oneself.

A fundamental question for theories in developmental science is how variations in parental values and practices influence their children. In older children, this may largely be driven by linguistic communication and explicit verbal teaching about values and identity^78–80^. In infants, we speculate that observational social learning plays a dominant role. Parental values “leak out” through their behavior. These behaviors are experienced first-hand by infants when parents engage with them^5,81^, and they can be learned through observation when infants see their parents interact with others. Research on imitation shows that infants watch, remember, and re-enact behaviors they see others perform^37^; infants also have the capacity to draw more abstract inferences about agents’ goals, dispositions, affiliations, and social evaluations, which cause infants to act in congruence^82–88^. As such, observational social learning may be a key mechanism by which parental life experiences, values, goals, and practices^89–91^ influence early prosocial tendencies. Because parental life experiences, values, goals, and practices differ across sociocultural groups, infants’ observational learning skills could help explain the sociocultural effects we observed in our experiments.

In conclusion, we found that infant *Homo sapiens* demonstrate significant possessive tendencies toward their own personal objects. Yet, some infants override this in favor of sharing by giving away treasured possessions as well as other categories of things. Infants did so in the absence of explicit rewards or immediate reciprocity by the non-kin stranger, despite having the desirable objects in their own hands and an immediate way for retaining possession. Interestingly, the expression of such behavior is related to sociocultural variables, suggesting that experiential factors may serve to calibrate the balance between possessive impulses and sharing behaviors during human infancy. The social-cognitive capacities that humans have in common with non-human primates, when coupled with rearing practices among human caregivers, may provide social learning experiences that enable infants to develop the uniquely *Homo sapiens* prosocial profile exhibited by adults.

## Methods

### Approval

The research was conducted with the approval of the University of Washington’s Human Subjects Institutional Review Board (Approval #00000832). All of the methods and procedures were performed according to the relevant guidelines and regulations. In all cases one of the parents of the infant participants provided informed consent.

### Participants

The participants were healthy, full-term infants from the Seattle metropolitan area. By design, all infants tested fell within a 1-month age range to reduce variance. A computerized subject pool run by the university was the source of participants. Soon after birth, parents were mailed an invitation for them to participate in infant studies. Parents who returned the card, or registered through a web interface, were later contacted through telephone and/or email to solicit participation. We used pre-established criteria for recruiting infants: normal birthweight (2.5–4.5 kg), normal length of gestational age (± 3 weeks of due date), and no known medical or developmental problems. The parking fees at the university laboratory site were reimbursed, and families received a small gift for participating.

The final analytic sample was *N* = 96 (581–611 days old). According to written responses on questionnaires provided to the parents, the racial-ethnic makeup of the final sample of infants was 87.50% White, 3.13% Asian, and 9.37% multiracial (indicated by two or more races); 6.25% of the infants were reported as being of Hispanic/Latino ethnicity. As in previous studies^28^, additional infants came to the university but were excluded from the final analytic sample using pre-established criteria. In Experiment 1, the exclusions were based on the following: extreme infant fussiness/tiredness/sickness (*n* = 3), experimental/equipment errors (*n* = 7), or interference by the parent or infant constant clinging to the parent (*n* = 8). For Experiment 2, the corresponding numbers for exclusions were: 6, 2, and 4.

In Experiment 1, the 48 infants (24 boys, 24 girls) ranged from 581 to 611 days (*M* = 19.60 months, *SD* = 0.28). In Experiment 2, the 48 infants (24 boys, 24 girls) ranged from 581–611 days (*M* = 19.53 months, *SD* = 0.29 months). This sample size was established prior to conducting the experiments. Based on published studies^28^, a power analysis (SAS software, Version 9.4) showed that a sample size of *N* = 40 per experiment (20 per group) would be sufficient to detect reliable differences, assuming a large effect size (*d* = 0.80) for the *P* value of 0.05 (two tailed) and a power of 0.80. The sample size was preset at *N* = 48 per experiment (24 per group) to satisfy counterbalancing.

### Test room, apparatus, and materials

The experiments took place at a university laboratory (270 cm × 190 cm) lined by blue curtains to provide a homogeneous environment relatively free of visual distractions. Test materials used in the studies included: (1) a tray (38.1 cm × 27.9 cm × 6.5 cm) onto which the objects were dropped, (2) the demonstration table (152 cm × 50 cm × 45 cm) with a black cloth skirt so that the participants could not see underneath it, (3) two blue cartons (38.5 cm × 38.5 cm × 78 cm) on either side of the table to divide the test room, and (4) a small gate (adjacent to one of the cartons), which the experimenter walked through to access the space behind the table and face the infant. This room setup was used to visually divide the room in two, with the infant and parent on one side of the table, and the adult experimenter on the opposite side of the table during the test period. The experimenter closed the gate after passing through it. This was done to show that the experimenter was in a separate area from the infant. The infant had direct access to their parent at all times; the parent sat quietly in a chair behind the infant during the test.

Three digital video cameras recorded the test session from different angles for subsequent coding. The experimenter camera was behind the infant and provided a view of the table area and the experimenter’s body; the infant camera was situated behind the experimenter and provided a view of the infant and the infant area, including the warm-up area with the parent; the overhead camera focused on a top-down view of the tray and the objects. The cameras were synched by a time generator which labeled time on a frame-by-frame basis (30 frames/s).

### Random assignment and counterbalancing

Infants were randomly assigned to the Experimental and Control group. We followed a rigorous procedure pertaining to the random assignment of infants: This assignment was made about 15 s before the experimental test began. After taking his place behind the table, the experimenter drew a card from a closed container which was kept below the table. This card specified the infant’s random assignment to group (Experimental vs. Control) and test order. In this way, there could be no bias in type/extent of play with the child before the experimental test, because the assignment was done only seconds before the test period began.

The order of object presentation was balanced within group in both experiments (*n* = 24 per group). For Experiment 1, two orders were used, which resulted in 12 infants (6 boys and 6 girls) allocated to each order within each group. Order 1 was bottle, toy; Order 2 was toy, bottle. For Experiment 2, six orders were used, which resulted in 4 infants (2 boys and 2 girls) assigned to each order within each group. The six orders were chosen using a Latin square design such that each object occurred equally often in each of the six positions. Order 1 was banana, block, bottle, grape, marker, favorite toy; Order 2 was block, bottle, banana, marker, favorite toy, grape; Order 3 was bottle, banana, block, favorite toy, grape, marker.; Order 4 was grape, marker, favorite toy, banana, block, bottle; Order 5 was marker, favorite toy, grape, block, bottle, banana; Order 6 was favorite toy, grape, marker, bottle, banana, block. After the study was completed, we found that one child was mistakenly run in order 3 instead of 6.

### Procedure

The design and procedure for the two experiments were essentially the same in that both involved the same groups and procedures, with the difference being that Experiment 1 had two trials (using two personal objects: favorite toy and bottle) and Experiment 2 had six trials (using two edible fruit: banana and grape; two common objects: block and marker; in addition to the two personal objects). We chose a slice of peeled banana and half of a juicy grape as the edible fruit because these were well-like foods by infants this age. In our previous study^28^, we asked parents to formally rate how much their infants liked this food using a Likert scale ranging from 1 (“strongly dislike) to 7 (“strongly like”). Results showed that both the banana and the grape were highly-liked fruits. The banana and grape were rated respectively as: mode = 7, *M* = 6.03; and mode = 7, *M* = 5.69.

The laboratory visit began by obtaining informed consent from the parent in a waiting room. Toward the end of consenting, the parent surreptitiously slipped the child’s objects into pouch and gave it to the experimenter. Next, the male experimenter walked the parent and infant to the separate testing room. Once there, the experimenter used a foot switch to turn on the cameras and said to the infant, “Look, there’s already some turtles waiting for us.” Small plastic turtles were visible near a bin of toys which were covered with a cloth. The experimenter asked the parent to sit in the back of the test room, and requested that the parent focus on a pen-and-paper questionnaire (pertaining to race, ethnicity, siblings, and whether or not infants slept with their favorite toy), so that the parents were not involved in the testing.

The experimenter then sat on the floor with the infant near the toy turtles, and removed the cloth to reveal a set of interesting toys in the bin (pairs of toy puppies, lions, cows, planes, whales, turtles, and plastic rings). Infants had the opportunity to warm up to the test environment and experimenter by playing with these toys. The warm-up was brief (*M* = 6.01 min, *SD* = 1.41) for both experiments. The adult experimenter encouraged the infant to play with the toy objects, verbally commenting on each object and showing specific actions that each afforded, such as shaking the plastic rings to make a noise, squeezing the rubber toys (lions, whales, turtles), and moving a toy part (dog feet/head, cow leg/head/tail, and plane wheels/propeller). The experimenter allowed the child to play until the (s)he had directed visual attention to all of the objects and had touched one or more. After this, the experimenter began to gather the objects to put them back into the bin and said that it was time for the toys to go to sleep as he covered the top of the toy bin.

The experimenter next opened the small gate so that he could walk behind the table to position himself for administering the test. The experimenter conspicuously closed the gate after passing through it. This procedure and gate were used to convey the impression that the experimenter was in a separate area from the infant. For theoretical reasons (described in the main text), we thought it important that the experimenter was spatially blocked from the infant by the table and gate, but that the infant had a direct and uninterrupted path to their parent, so that infants could easily grab the dropped item and retreat to their parent if desired.

To attract the infants’ attention to the test table, the experimenter put a squeaky, yellow rubber duck on one corner of the table. After the infant visually fixated it, the experimental procedure was launched. The first act was for the experimenter to draw a card from a box that was kept out of view below the table. The card specified the random assignment of the subject to the Experimental or Control Group. (These random assignment cards were managed by a research assistant to ensure that counterbalancing was achieved. This procedure ensured that the experimenter was unaware of the infant’s group assignment until the experimental procedure began, reducing potential experimental bias during warm-up.) At that point, the experimenter launched the test by announcing, “I am going to show you some things.”

In Experiment 1, each infant was administered two trials, one with each object. Each trial consisted of the experimenter taking an object from below the table and holding it up in front of him and excitedly noting the presence of the object to attract the infant’s attention (see verbal script below). From there the procedure branched to follow one of two paths according to the infant’s randomly assigned group. In Experiment 2, each infant was administered six trials, one with each object. Other than this change, the rest of the procedure followed was the same as described in Experiment 1.

In the Experimental Group, the adult experimenter (*E*) fumbled the objects such that they “accidentally” fell out of his hand and landed out of his reach onto a tray, which was on the floor on the infant’s side of the table (rarely some objects fell on the floor). An electronically timed 20-s response period was used, starting from the moment the object contacted the tray (or the floor). In the first 10 s of this period, the *E* unsuccessfully reached for the object; for the next 10 s, the *E* continued his reaching efforts as he alternately looked back and forth between the object and the subject. After each 20-s trial, whether or not the subject handed the object to the *E*, he said “Ah, interesting.” In the Control Group, the *E* also brought up an object from below the table. The *E* then appeared to intentionally aim and gently toss the object onto the tray and then rested his hands on the table. As in the Experimental Group, the 20-s response period was timed, and at the end of each trial, regardless of whether the infant gave him the object, the *E* simply said “Ah, interesting.”

For both of the experiments, the verbal script used by the experimenter, when he initially brought an object from below the table into view, was identical for both groups. In Experiment 1, for the first trial the experimenter said, “Here it is! See it?” (The favorite objects were not named as these were expected to vary widely between infants.) After the first trial ended and to introduce the second trial, the experimenter said, “Guess what, there’s something else.” To begin the second trial, when the experimenter brought the object into view he again said, “Here it is. See it?” In Experiment 2, for the common object trials, the experimenter said “It’s a block. See it?” or “It’s a marker. See it?” For the edible object trials, the experimenter said “It’s a banana. See it?” or “It’s a grape. See it?” For the personal object trials, the experimenter said, for both trials, “Here it is. See it?” Between each trial, the experimenter provided a transition. Transition 1 was “And…There’s something else!”; Transition 2 was “Whoa…There’s something else!”; Transition 3 was “Guess what…There’s something else!”; Transition 4 was “Wow…There’s something else!”; Transition 5 was “Oh my…There’s something else!”.

### Behavioral scoring

The infant behavior during the 20-s response period was scored from the video records, primarily based on the camera facing the infant, but if the target object or infant’s arm was obscured, the other two camera angles were available to coders to obtain the best view of the response. The coders were unaware of the experimental hypotheses. During the response period for each trial, the behavior scored was the presence or absence of infant giving, i.e., whether or not the infant placed the object on the experimenter’s hand. During the demonstration period for each trial, the coder scored the presence or absence of each of three types of possessive behaviors, i.e., whether infants: (1) prepared to grasp the object with one hand extended toward object with hand shaping/alignment, (2) demonstrated an approach effort toward the object by rising onto the balls of the feet (both heels off floor) to lean or lunge toward the object, (3) raised both arms toward the goal object. The demonstration period was defined as the interval between when the experimenter first showed the object (experimenter’s hand brought the object up from below the table edge) to his relinquishment of the object from his hand (so that it dropped to the tray on floor). The main coder scored all videos and a second coder scored a randomly selected 25% of them. Coders achieved good to excellent agreement on all measures as assessed by Cohen’s kappa. For the measure of giving the object to the adult, there were no disagreements on either intra- or interscorer assessments (κ = 1.00). For the three measures of infant possessive behaviors, the intrascorer kappas were 0.98, 1.00, and 0.93; for the interscorer kappas were 0.79, 0.74, 0.85.

### Background and classification variables

For the sibling and ethnic-cultural background variables, infants were classified following the same procedure described elsewhere^28^. Specifically, parents completed a pen-and-paper questionnaire that asked about their infant’s siblings, race, and ethnicity. Based on these responses, infant participants were classified according to whether they did or did not have siblings. The options provided to parents for their infant’s race/ethnicity followed the guidelines from the U.S. National Institutes of Health (NIH), Parents indicated their infant’s race (by selecting all that applied): American Indian or Alaskan Native, Asian, Black/African-American, Native Hawaiian or Other Pacific Islander, White. In a separate item, parents classified their infant as Hispanic/Latino ethnicity or not. Research in the field of social psychology has grouped people from Asian and Hispanic/Latino background as being from what is termed “interdependent cultures”^45,46^; we followed this approach and grouped them together in order to derive the ethnic-cultural background variable. Pooled with our prior work^28^, 192 infants were sorted according to whether they had siblings (*n* = 67) or not (*n* = 125), and whether parents reported Asian and/or Hispanic/Latino background (*n* = 18) or not (*n* = 174). For the classification of a different variable concerning infants’ home behavior with their favorite toy, participants were sorted according to whether or not they slept with their favorite toy based on information from the parental questionnaire.

## Supplementary Information

Supplementary Information

## Data Availability

The data that support the findings of these studies are available from the corresponding authors on reasonable request.
