# Supplementary material for: Human infants can override possessive tendencies to share valued items with others
Source: Sci Rep. 2021 May 5;11:9635. doi: 10.1038/s41598-021-88898-x (PMC8100139; doi:10.1038/s41598-021-88898-x)
Supplement: Supplementary file 1 — Supplementary Information [file 41598_2021_88898_MOESM1_ESM.pdf]

Supplementary Information

Human infants can override possessive tendencies to share valued items with others

Rodolfo Cortes Barragan[1,2*], Andrew N. Meltzoff[1,2*]
[1]Institute for Learning & Brain Sciences, University of Washington
[2]Department of Psychology, University of Washington
*Correspondence to: barragan@uw.edu; meltzoff@uw.edu

Related research with human infants

We wish to acknowledge two prior studies that inform the issues investigated in this paper, albeit in ways that differ from the approach used in this paper in important ways. In one previous study[1] the researchers presented 18-month-old infants with multi-step scenarios in which infants could provide help with their items brought from home or designated for them in the laboratory. For example, in one scenario, the experimenter placed the child's blanket from home on the tray and mentioned that the blanket must be warm, and then the experimenter became cold, and "suddenly becomes cold, shivering, rubbing her arms, and saying 'Brrrr' while looking distressed" (p. 1819-1820). In another scenario, after placing the child's toy brought from home on a tray and telling the child that their toy must make them happy, the experimenter received a phone call that made her sad, and the question was whether the infant gave his/her own toy to offset this sadness. In a third scenario, infants were first given a hairclip and told it would be for them, and at test the experimenter needed a hairclip to fix her messy hair which was making her frustrated. A key feature in all three of the tested scenarios is that children were provided with a series of increasingly explicit cues to give the object to the experimenter (up to 8 stages), including starting with the communication of a need (e.g., shivering while looking distressed), continuing with verbal statements of need (e.g., "I need something to make me warm"), proceeding to a verbal request for help ("Can you help me?") and culminating in a specific verbal request that the child could fulfill (e.g. "Can you bring me the blanket?"). The results showed that some infants gave objects (blanket, toy, hairclip) to the adult especially during the stages that used explicit verbal requests. In a related study that used the same multi-step response period with linguistic instructions[2], the experimenter gave mothers of 16- to 36-month-olds items for at-home use by the child, e.g., a scarf. A week later in the test, the experimenter assessed children's willing to give these items to the experimenter when they witnessed a similar 7-stage series of increasingly explicit cues similar to that used in the prior study, e.g. "Can you give me the scarf?" (Supplementary Online Materials, p. 8). It is not entirely clear that a one-week exposure period constituted sufficient time for the assigned object (e.g., a scarf) to have become a high-valuable or treasured object for infants. Also of note is that the age range tested was quite wide, up to 36 months of age, and the older children would be expected to understand and follow the explicit linguistic instructions to "give me the scarf." In this sense the study, although highly relevant, may not test the same point as addressed in our current work.

References

1. Svetlova, M., Nichols, S. R. & Brownell, C. A. Toddlers' prosocial behavior: From instrumental to empathic to altruistic helping. *Child Dev*. **81**, 1814-1827 (2010).
2. Corbit, J., Callaghan, T., Svetlova, M. Toddlers' costly helping in three societies. *J. Exp. Child Psychol*. **195**, 104841 (2020).
